# Acute kidney injury in children with COVID-19: a retrospective study

**DOI:** 10.1186/s12882-021-02389-9

**Published:** 2021-05-31

**Authors:** Jameela Abdulaziz Kari, Mohamed A Shalaby, Amr S Albanna, Turki S Alahmadi, Adi Alherbish, Khalid A Alhasan

**Affiliations:** 1 Pediatric Nephrology Center of Excellence, Department of Pediatrics, King Abdulaziz University, PO Box: 80215, 21589 Jeddah, Kingdom of Saudi Arabia; 2 King Abdullah International Medical Research Center, King Saud Bin Abdulaziz University for Health Sciences, Jeddah, Kingdom of Saudi Arabia; 3 Department of Pediatrics, King Abdulaziz University and Faculty of Medicine in Rabigh, Jeddah, Kingdom of Saudi Arabia; 4 Pediatrics Department, College of Medicine, King Saud University, Riyadh, Kingdom of Saudi Arabia

**Keywords:** Acute Kidney Injury, COVID-19, Child, Multisystem Inflammatory Syndrome in Children

## Abstract

**Background:**

Acute kidney injury (AKI) is a complication of coronavirus disease 2019 (COVID-19). The reported incidence of AKI, however, varies among studies. We aimed to evaluate the incidence of AKI and its association with mortality and morbidity in children infected with severe acute respiratory distress syndrome coronavirus 2 (SARS-CoV-2) who required hospital admission.

**Methods:**

This was a multicenter retrospective cohort study from three tertiary centers, which included children with confirmed COVID-19. All children were evaluated for AKI using the Kidney Disease Improving Global Outcomes (KDIGO) definition and staging.

**Results:**

Of 89 children included, 19 (21 %) developed AKI (52.6 % stage I). A high renal angina index score was correlated with severity of AKI. Also, multisystem inflammatory syndrome in children (MIS-C) was increased in children with AKI compared to those with normal kidney function (15 % vs. 1.5 %). Patients with AKI had significantly more pediatric intensive care admissions (PICU) (32 % vs. 2.8 %, *p* < 0.001) and mortality (42 % vs. 0 %, *p* < 0.001). However, AKI was not associated with prolonged hospitalization (58 % vs. 40 %, *p* = 0.163) or development of MIS-C (10.5 % vs. 1.4 %, *p* = 0.051). No patient in the AKI group required renal replacement therapy. Residual renal impairment at discharge occurred in 9 % of patients. This was significantly influenced by the presence of comorbidities, hypotension, hypoxia, heart failure, acute respiratory distress, hypernatremia, abnormal liver profile, high C-reactive protein, and positive blood culture.

**Conclusions:**

AKI occurred in one-fifth of children with SARS-CoV-2 infection requiring hospital admission, with one-third of those requiring PICU. AKI was associated with increased morbidity and mortality, and residual renal impairment at time of discharge.

## Background

Coronavirus disease 2019 (COVID-19) is a new disease that emerged in China in late 2019 [1]. Since then, it has caused an unprecedented health, social, and economic crisis. Similar to other coronaviruses, patients with COVID-19 usually present with respiratory and gastrointestinal symptoms, but more severe cases progress to a systemic disease that affects almost all organs [2, 3].

Acute kidney injury (AKI) is a complication of COVID-19 and the pathophysiology of AKI in COVID-19 appears to be multifactorial. The direct viral causes of AKI in COVID-19 are hypothesized as direct viral injury, dysregulated inflammation with cytokine storm, and vascular injury [4]. However, other factors found in similar diseases have also been described, including hypovolemia, heart failure (both right and left), sepsis, and dehydration [5, 6].

The exact prevalence of AKI in patients with COVID-19 is unknown. Initial reports from China reported a low prevalence of 0.5 % [7]; however, in a later study, the prevalence rose to 19 % [8]. Over time, and with a highly scrutinized data collection, the prevalence of AKI appears to be even higher. In two large multicenter studies conducted in New York, AKI was reported in 22.2 and 36.6 % of 5700 patients (adults and children) and 5449 (adult only) patients, respectively [9, 10]. In another cohort, AKI was reported in 46 % of 3235 admitted patients [11]. The latter article reported that 68 % of all critical care patients had AKI, and 20 % of all admitted patients needed renal replacement therapy (RRT).

Similarly, the reported epidemiology of AKI in children infected with severe acute respiratory distress syndrome coronavirus 2 (SARS-CoV-2) is variable. In a cohort of 52 children in the UK, 46 % had a serum creatinine level above the normal upper limit and 29 % met the definition of AKI, but none required RRT [12]. In another multicenter study, 18 % of critically ill children with COVID-19 had AKI [13]. These two studies reported a strong association between AKI and Multisystem Inflammatory Syndrome in Children (MIS-C). This is a severe form of COVID-19 presentation that usually occurs a few weeks after the initial (usually milder) symptoms [13].

In this multicenter study, we examined data from all children admitted with COVID-19 to estimate the prevalence of AKI and its associated risk factors, as well as the required care of those children.

## Methods

This is a multicentric retrospective cohort study from 3 tertiary centers (King Abdulaziz University Hospital, King Khalid University Hospital, and East Jeddah General Hospital) in the Kingdom of Saudi Arabia (KSA).

### Inclusion criteria

All children who were positive for COVID-19, determined by polymerase chain reaction (PCR) of nasal swab samples and requiring hospital admission at one of the participating centers between March 1st and mid-July 2020, were included. Children were defined as ≤ 14 years old. All included patients were considered ill because of the required hospital admission. All participating centers used the same visual triage checklist for acute respiratory infection in suspected cases to determine who needed screened using a nasopharyngeal swab.

All patients labeled as positive for COVID-19 had their nasopharyngeal and\or endotracheal swab samples screened by PCR. Patients were considered infected if the initial swab result was positive, or if it was initially negative with a positive repeat test. Only the first admission was considered for patients who had multiple hospital admissions.

### Exclusion criteria

We excluded positive COVID-19 patients with insufficient data, those who were seen in the emergency department with positive swabs but did not require admission, or any patient with a previous admission for COVID-19. We also excluded neonates (defined as those aged < 28 days at presentation) and patients with evidence of preexisting chronic kidney disease stages III–V.

### Data collection

 Clinical and laboratory data were collected and merged from electronic health records of all participating centers. Data included patient demographic information (age, gender, and nationality), anthropometric measures and centile, presenting symptoms and signs, associated comorbidities, and need for pediatric intensive care unit (PICU). For clinical information, we examined the severity of respiratory distress, blood pressure interpretation, oxygen saturation, need for oxygen therapy, and mechanical ventilation.

Renal function assessment was performed using the following data: baseline creatinine, baseline estimated glomerular filtration rate (eGFR), highest creatinine, and lowest eGFR recorded during the initial 7 days of admission. We calculated urine output (UOP) in children with available UOP data. We identified patients with AKI using the Kidney Disease Improving Global Outcomes (KDIGO) consensus definition [14]. KDIGO defines AKI as an increase in serum creatinine by ≥ 0.3 mg/dL (≥ 26.5 µmol/L) within 48 h; OR an increase in serum creatinine ≥ 1.5 times the baseline level within the prior 7 days; OR a urine volume ≤ 0.5 mL/kg/hour for the first 48 h of admission (for patients who had UOP information). We categorized AKI patients into 3 main stages. Stage I consisted of patients with an increase in serum creatinine by ≥ 0.3 mg/dL (≥ 26.5 µmol/L), or an increase in serum creatinine to ≥ 1.5 times the baseline level within the prior 7 days, and/or a UOP of ≤ 0.5 mL/kg/hour over 6–12 h. Stage II patients had an increase in serum creatinine 2 to 2.9 times from the baseline level and/or a decrease in UOP of ≤ 0.5 mL/kg/hour for 12 to 24 h. Stage III patients had an increase in serum creatinine by ≥ 353.6 mmol\L or greater than 3 times the baseline level, a decrease in GFR to < 35 ml\min\1.73 m2, and/or a decrease in UOP of ≤ 0.5 mL/kg/hour for more than 24 h.

We also calculated the renal angina index (RAI = 1–40) for all participants using the multiplication of both risk level and injury level. A score ≥ 8 is considered a positive RAI [15]. Estimated glomerular filtration rate was calculated using the modified Schwartz formula [16]. Baseline creatinine was defined as the last creatinine within the previous 6 months prior to the PICU. For those patients admitted for the first time, we used an average GFR according to age, sex, and height of the child [17].

For any patient that required ICU admission, we calculated the Pediatric Risk of Mortality (PRISM version IV) during the first 24 h of ICU admission [18].

All laboratory workups were recorded and followed for identification of significantly associated hematological and\or laboratory abnormalities, such as significant anemia, leucopenia or leukocytosis, neutropenia or neutrophilia, thrombocytopenia, and disturbed coagulation profiles. Other associated laboratory abnormalities, such as hypernatremia, hyperkalemia, and high troponin levels were reported. Evidence of any associated infection (blood and urine culture) and the high acute phase reactants (C-reactive protein and ferritin) were recorded and highlighted.

Clinical outcome indicators included mortality rate, abnormally high creatinine, high blood pressure, or proteinuria.

### Definitions

Significant exposure to nephrotoxic medications (nephrotoxic injury negated by just-in-time action = NINJA score): exposure to 3 or more common nephrotoxic medications for 1 day or to aminoglycosides for 3 or more days [19].

Hypertension: 2 or more systolic and diastolic blood pressure readings at 2 different time points > 95th percentile for gender, age, and height.

Hypotension: 2 or more systolic and diastolic blood pressure readings at 2 different time points < 50th percentile for gender, age, and height.

Hyperkalemia: serum potassium level > 5.5 mmol\L.

Hypernatremia: serum sodium level > 146 mmol\L.

Anemia: a reduction of the hemoglobin concentration to 2 standard deviations below the mean, based on age-specific normal levels.

Leukocytosis and leucopenia: total leucocytic count higher than the mean normal value based on age.

Neutropenia: a neutrophilic count < 500/mm^з^.

Oliguria: a UOP < 0.5 ml\kg\hr.

We used the World Health Organization definition for MIS-C and adolescents aged 0–19 years. Their criteria were fever for 3 days or more and two of the following: skin rash or signs of mucocutaneous inflammation hypotension or shock, features of myocardial dysfunction, evidence of coagulopathy, acute gastrointestinal problems, and elevated markers of inflammation, such as erythrocyte sedimentation rate, C-reactive protein, or procalcitonin, with evidence of COVID-19 and no other obvious microbial cause of inflammation [20].

 The study was approved by the Biomedical Ethics Research Committees of the three recruiting centers (King Abdulaziz University –faculty of medicine, King Khalid University – medical college, and East Jeddah General Hospital,). Consent from participants was not required as this was a retrospective study using data collected for routine clinical practice.

### Statistical analysis

All analyses were performed using STATA (StataCorp. 2011. Stata Statistical Software: Release 12. College Station, TX). The proportion and mean for dichotomous and continuous variables, respectively, were measured to describe a patient’s characteristics. The association between AKI development and different outcomes (prolonged hospitalization, ICU admission, and mortality) was measured using the chi-square test. Multiple regression analysis was also performed to adjust for age, sex, and the presence of comorbidities. Predictors of residual renal impairment at discharge was measured using the chi-square test. Statistical significance was determined using a 95 % confidence interval and a *p*-value of 0.05.

## Results

Eighty-nine children admitted with a confirmed diagnosis of COVID-19 were included in this study. Of these,19 (21 %) developed AKI upon admission. Patients with AKI were younger than those with normal kidney function. Most of the children in both groups tended to be overweight with a median body mass index in the 91st percentile. Children with AKI had an increased number of comorbidities compared to patients with normal kidney function (Table 1).

**Table 1:** Baseline patient demographics and disease characteristics

Characteristics	Acute Kidney Injury (No. 19)		Normal Renal Function (No. 70)		*P* Value
	Estimate	95 % CI	Estimate	95 % CI	
Age (mean, months)	54.6	30.0–79.3	74.7	61.1–88.3	0.1676
(median, months)	24	11.5–111.4	72	36.0–92.7	
Male sex (%)	47.4	24.4–71.1	40.0	28.5–52.4	0.563
Saudi nationality (%)	47.4	24.4–71.1	54.3	41.9–66.3	0.592
Length (mean, cm)	85	72–99	107	98–115	0.0140
(median, cm)	80	66.6–101	104	93.3–117	
(mean, percentile)	9.7	0–21.1	37.2	27.7–46.8	
(median, percentile)	0.4	0.3–2	25	2.0–50.0	
BMI (mean kg/m^2^)	19.4	16.1–22.8	20.8	18.7–22.9	0.5296
(median kg/m^2^)	17.9	14.7–22.2	18.9	16.4–22.2	
(mean, percentile)	61.3	41.4–81.2	66.2	56.3–76.2	
(median, percentile)	91	20.1–98.5	91.0	75.0–98.0	
Contact with COVID patients	78.9	54.4–93.9	82.9	72.0–90.8	0.694
Recent travel	10.5	1.3–33.1	4.3	0.9–12.0	0.295
Comorbidities^a^ (%)	63.2	38.4–83.7	18.6	10.3–29.7	< 0.001

*Abbreviations*: *BMI *body mass index, *CI *confidence interval, *cm *centimeter

Notes: ^a^Comorbidities include diabetes, renal disease, respiratory disease, cardiac disease, malignancy/hematological disorders, and/or metabolic disorder

**Fig. 1:**
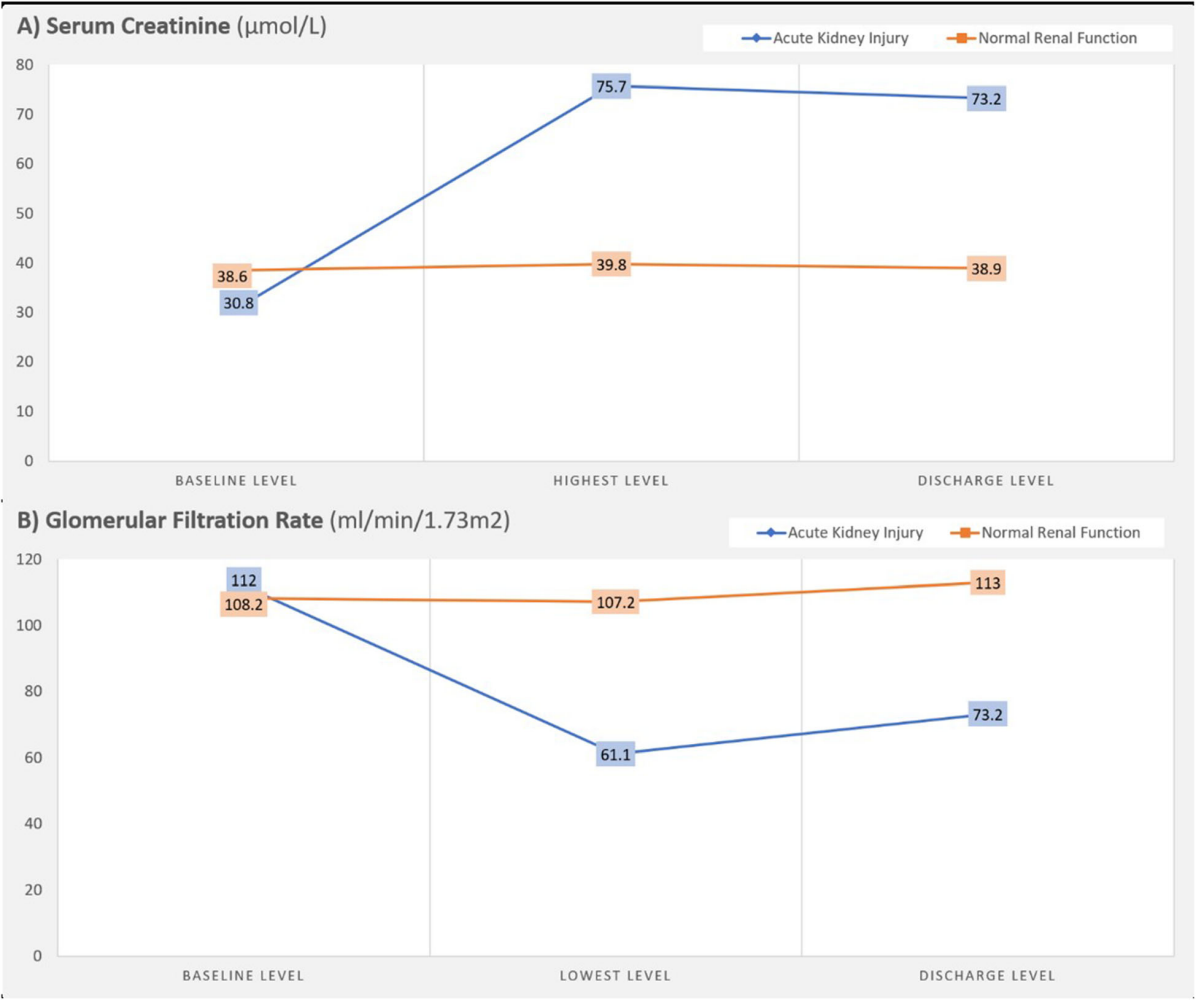
Serum creatinine and glomerular filtration rate changes among COVID-19 children admitted with and without acute kidney injury

None of the admitted patients had chronic renal impairment and had almost normal baseline levels of creatinine and eGFR. Figure 1 shows the average change in serum creatinine and eGFR levels during admission of patients who did and did not develop AKI.

Using KDIGO staging for AKI, 58 % of patients with AKI had stage I, 31.5 % had stage II, and 10.5 % had stage III. Their RAI score was < 8 (insignificant) in 10 out of the 11 children with stage I AKI. However, all children with AKI stages II and III had a significant RAI score (≥ 8), and all children with stage III AKI had a maximum RAI score of 40 (Table 2).

**Table 2:** Parameters related to nephrology assessment of COVID-19 children admitted with acute kidney injury (19 children)

	**Status of renal function**		
	**Normal (70 children)**	**KDIGO I (11 children)**	**KDIGO II & III (8 children)**
RAI (number (%))			
Score <8 (non-significant)	70(100%)	10 (90.9)	0
Score 8	0	1 (9.1)	5 (62.5)
Score 40	0	0	3 (37.5)
NINJA-positive (number (%))	9 (12.9)	1 (9.1)	7 (87.5)
Time to worsening of renal function^a^ (mean (SD), hrs.)	-	12.8 (13.8)	6 (8.6)
Nephrotoxic drug administration (number (%))			
None	60 (85.7)	9 (81.8)	1 (12.5)
One drug	5 (7.1)	2 (18.2)	4 (50)
Multiple drugs	5 (7.1)	0	3 (37.5)
Duration of nephrotoxic therapy (mean (SD), days)	6.1 (3.5)	3.5 (2.1)	5 (2.4)
Nephrology referral (number (%))	0	0	3 (37.5)
Oliguria (number (%))	0	0	1 (12.5)
Hypervolemia (number (%))	0	0	0
Use of RRT (number (%))	0	0	0
Residual renal impairment at discharge (number (%))	0	3 (27.3)	5 (62.5)

*Abbreviations*: *KDIGO* Kidney* Disease Improving Global Outcomes*, *RAI* renal angina index, *NINJA* nephrotoxic injury negated by just-in-time action, *SD* standard deviation; *RRT* renal replacement therapy

Notes: ^a^Time from admission to deterioration of renal function

**Fig. 2:**
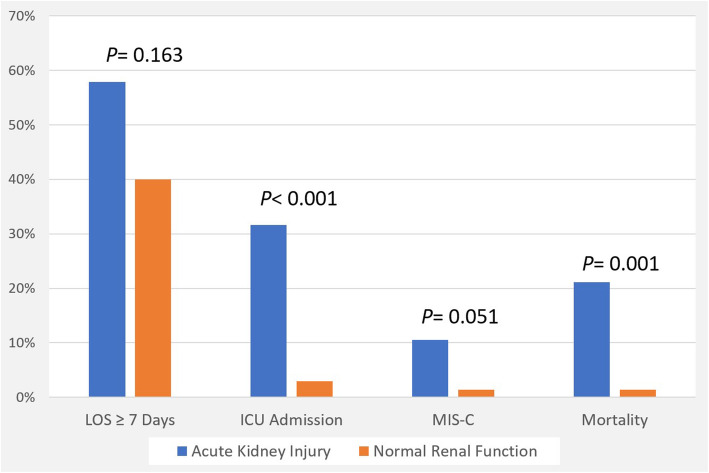
COVID-19 related clinical outcomes associated with acute kidney injury

15 % of AKI patients (all with stage III AKI) presented with the full diagnostic criteria of MIS-C, compared to only 1.5 % of non-AKI children (Fig. 2). Furthermore, renal function deteriorated earlier in children with stage II and III compared to children with stage I AKI. The NINJA indicator of significant exposure to nephrotoxic medications was more frequently positive in patients with stage II and III AKI (87.5 %) than in those with either stage I AKI (9.1 %) or normal renal function (12.9 %) (*p* < 0.001). Most patients with AKI (95 %) were non-oliguric and there was no evidence of hypervolemia in any patient. RRT was not prescribed for any patient with AKI (Table 2).

AKI was significantly associated with more frequent ICU admission (32 % vs. 2.8 %, *p* < 0.001) and mortality (42 % vs. 0 %, *p* < 0.001), but not with prolonged hospitalization (58 % vs. 40 %, *p* = 0.163) or development of MIS-C (10.5 % vs. 1.4 %, *p* = 0.051) compared to patients with normal kidney function (Fig. 2). After adjustment for age, sex, and the presence of comorbidities, AKI was still significantly associated with mortality (adjusted OR: 14.6, *p* = 0.041) and ICU admission (adjusted OR: 10.1, *p* = 0.009). Residual renal impairment at discharge occurred in 9 % of our population, which was influenced by the following predictive factors: presence of comorbidities (*p* = 0.023), hypotension (*p* < 0.001), hypoxia (*p* = 0.02), heart failure (*p* = 0.001), adult respiratory distress syndrome (ARDS) (*p* = 0.005), hypernatremia (*p* = 0.011), abnormal liver profile (*p* = 0.046), high C-reactive protein (*p* = 0.033), and positive blood culture (*p* = 0.002) (Table 3).

**Table 3:** Factors associated with residual renal impairment at discharge of COVID-19 children

Factors		Residual Renal Impairment^a^ (%)	*P* Value
Age	≥ 5 years	10.4	0.350
	< 5 years	5.0	
Sex	Male	8.1	0.806
	Female	9.6	
Nationality	Saudi	10.6	0.565
	Non-Saudi	7.1	
Comorbidities^b^	No	4.7	**0.023**
	**Yes**	**20.0**	
Dyspnea	No	9.1	0.966
	Yes	8.8	
GI symptoms^c^	No	9.2	0.895
	Yes	8.3	
Skin rash	No	8.1	0.134
	Yes	33.3	
Arterial BP	Normal	6.9	**< 0.001**
	High	0	
	**Low**	**75.0**	
Hypoxia	No	5.6	**0.020**
	**Yes**	**23.5**	
Dehydration	No	8.2	0.588
	Yes	12.5	
Heart failure (clinical signs)	No	8.0	**0.001**
	**Yes**	**100**	
Chest radiograph	Normal	7.6	**0.005**
	Mild Pneumonia	5.6	
	Severe Pneumonia	0	
	**ARDS**	**66.7**	
Anemia	No	7.0	0.386
	Yes	12.5	
WBC	Normal	4.0	0.164
	Low	18.2	
	High	14.3	
Serum sodium	Normal	5.3	**0.011**
	Low	25.0	
	**High**	**33.3**	
Hyperkalemia	No	9.6	0.425
	Yes	0	
LFT	Normal	6.0	**0.046**
	**Abnormal**	**21.1**	
CRP	Normal	3.8	**0.033**
	**High**	**17.1**	
Troponin I	Normal	9.4	0.262
	High	20.0	
Blood culture	Negative	6.8	**0.002**
	**Positive**	**42.9**	

*Abbreviations*: *GI *gastrointestinal, *BP *blood pressure, *ARDS *acute respiratory distress syndrome, *WBC *white blood cells, *LFT *liver function test, *CRP *C-reactive protein

Note: ^a^Abnormal renal function at the time of discharge. ^b^ Comorbidities include diabetes, renal disease, respiratory disease, cardiac disease, oncology/hematology disorders, and/or metabolic disorder. ^c^Diarrhea or vomiting

## Discussion

The emergence of COVID-19 in December 2019 prompted many pediatric nephrology units to prepare for an increased demand for dialysis and PICU services. It has been determined that children seem to be less severely affected by this virus than adults. The surge in hospitalized adult patients requiring dialysis caused many resources previously allocated to pediatrics to be diverted to adults. Position statements and articles were published to inform pediatric nephrologists about the proper utilization of their resources [5, 21]. Nonetheless, an exact estimate of the prevalence of AKI in children hospitalized with COVID-19 remains essential to guide such decisions.

Our study examined eighty-nine children who were hospitalized with COVID-19. Of these, 21 % developed AKI, which is lower than previously reported by Stewart et al. in their cohort of 52 children admitted to the Great Ormond Street Hospital [12]. In their report, approximately 29 % of children developed AKI. The discrepancies between our findings and those of Stewart et al. could be due to the different definitions of AKI used. Of note is that Stewart et al. used the British Association of Pediatric Nephrology (BAPN) diagnostic criteria to define AKI. Another factor that may explain the lower prevalence of AKI in our cohort could be the lower incidence of MIS-C. In our cohort, only 15 % of AKI patients met the full diagnostic criteria of MIS-C associated with SARS-Cov-2, while in the study by Stewart et al., 73 % of those with AKI met the criteria of MIS-C associated with SARS-Cov-2 [22]. The incidence of MIS-C was marginally higher, albeit not significantly higher, when compared with that in children with normal kidney function (p = 0.051). This could be explained by the small number of children analyzed.”

One of the most remarkable aspects of AKI in this study is its asymptomatic nature. In our patients, 95 % had no oliguria, none developed hypervolemia, and none required RRT. A decline in mean GFR reached 61.1 ml/min/1.73 m^2^ at its lowest, a level not known to cause considerable electrolyte or volume disturbances. The time to worsening renal function was shorter in patients with stage II and III AKI, reaching a mean of 6 h. Of note is that the RAI score was high in all cases of stage II and III AKI, and a negative score was found in 90 % of stage I patients. Despite the asymptomatic nature of AKI in this cohort, its early identification and prevention is important considering its association with higher morbidity and mortality. The association between AKI and PICU admission could be explained by a worsening general condition and hemodynamic instability necessitating ICU admission, and hence an increasing incidence of AKI. Nonetheless, AKI cannot be excluded as the sole cause of increased PICU admissions, especially in its more severe forms [23, 24]. Presence of comorbidities was important in those who developed AKI and those who ended with residual renal impairment at discharge. Hence, it is important to follow UOP and creatinine carefully in children infected with SARS-CoV-2, especially if they have comorbid conditions, such as respiratory diseases, cardiac diseases, hematological disorders, renal diseases, diabetes, and malignancies. There was also a tendency for AKI to happen at a relatively younger age.

As stated above, 9 % of our study population developed residual renal impairment at the time of discharge. Factors associated with residual renal impairment were either related to decreased tissue perfusion (hypotension, heart failure, hypoxia, acute respiratory syndrome), sepsis (high C-reactive protein, positive blood culture), worsening clinical condition (hypernatremia, increased liver enzymes), or comorbidities. What intrigued us was the failure of dehydration to reach statistical significance despite it being reported as an important culprit of AKI caused by SARS-CoV-2, a disease known to cause diarrhea in 8.1 % of children [22]. This can be explained by the possibility of rapid detection and the early correction of dehydration before it contributed to an increased risk of residual renal impairment.

One of the limitations of this study is its small population size. Fortunately, children infected with SARS-CoV-2 do not need to be hospitalized as frequently as adults, an obstacle interfering with all pediatric epidemiological studies in COVID-19. We attempted to overcome this limitation by forming a collaboration between three pediatric hospitals, reaching a cohort of 89 children. Another limitation is the retrospective nature and short duration of this study. It would be interesting to follow those who had residual renal impairment at the time of discharge to see whether it improves with time or translates into permanent chronic kidney disease.

## Conclusions

In conclusion, in the setting of COVID-19, AKI occurred in approximately one-fifth of our hospitalized children, and more than one-third of those required PICU admission. AKI is more commonly found in younger children and in those with comorbid conditions. AKI is associated with increased mortality and morbidity. A small proportion of children with AKI can develop residual renal impairment at the time of discharge. Nonetheless, it tends to be milder than in adults, with a lower incidence of oliguria and less need for RRT.

## Data Availability

The datasets used and/or analysed during the current study available from the corresponding author on reasonable request.”

## Abbreviations

ARDS: Acute respiratory distress syndrome
COVID-19: Coronavirus disease 2019
eGFR: Estimated glomerular filtration rate
KDIGO: Kidney Disease Improving Global Outcomes
LFT: Liver function test
MIS-C: Multisystem inflammatory syndrome in children
NINJA: Nephrotoxic injury negated by just-in-time action
PCR: Polymerase chain reaction
PICU: Pediatric intensive care admissions
RAI: Renal angina index
RRT: Renal replacement therapy
UP: Urine output
